# Effects of one long vs. two short resistance training sessions on training volume and affective responses in resistance-trained women

**DOI:** 10.3389/fpsyg.2022.1010596

**Published:** 2022-09-29

**Authors:** Helene Pedersen, Atle Hole Saeterbakken, Marius Steiro Fimland, Vegard Moe Iversen, Brad J. Schoenfeld, Nicolay Stien, Vidar Andersen

**Affiliations:** ^1^Faculty of Education, Arts and Sports, Western Norway University of Applied Sciences, Sogndal, Norway; ^2^Department of Neuromedicine and Movement Science, Faculty of Medicine and Health Sciences, Norwegian University of Science and Technology, Trondheim, Norway; ^3^Unicare Helsefort Rehabilitation Centre, Rissa, Norway; ^4^Department of Health Sciences, Lehman College, New York, NY, United States

**Keywords:** RPE, RPD, sPDF, EES, perception, training frequency

## Abstract

The aim of this study was to compare the acute effects of performing a lower body resistance training program in one long or two shorter sessions in 1 day on training volume and affective measures. Employing a randomized-crossover design, 23 resistance-trained women (22 ± 2 years, 166 ± 6 cm, and 66.4 ± 7.5 kg) performed two training days consisting of (i) one long (46 min) or (ii) two short sessions (total of 43 min) separated by 3.5–5 h. Each training day was separated by 4-6 days and consisted of three sets to failure for six exercises. Training volume (number of repetitions lifted) were recorded during the sessions. Rating of perceived exertion for effort (RPE), rating of perceived exertion for discomfort (RPD), session displeasure/pleasure (sPDF) and exercise enjoyment (EES) were measured 10 min after each session. Participants also completed a readiness to train questionnaire (7 questions), 24 h after each session, and which training protocol they preferred, 48 h after the last session. The long session led to higher RPE (+1 point, *p* < 0.001, ES = 1.07), RPD (+1 point, *p* = 0.043, ES = 0.53) and sPDF (*p* = 0.010, ES = 0.59) compared to the short sessions. There was no difference in EES (*p* = 0.118, ES = 0.33). The short sessions had 3% higher training volume than the long session (*p* = 0.002, ES = 0.42). There were no differences in perceived readiness to train 24 h after the sessions (range: *p* = 0.166–0.856 and ES = 0.08–0.32). Twenty-two participants preferred the long session, while one preferred the short sessions. In conclusion, performing a longer, lower body, resistance training session led to greater perceptions of effort, discomfort and session pleasure than splitting the same program into two shorter sessions among resistance-trained women. However, two shorter sessions led to a greater training volume.

## Introduction

Training frequency is typically defined as the total number of weekly resistance training sessions and is one of several components to consider when designing resistance training programs (Bird et al., 2005; ACSM, 2009). Performing briefer, more frequent sessions could potentially allow for increased training load or more repetitions lifted at same training load compared to longer and less frequent sessions due to reduced fatigue and higher energy utilization (Hartman et al., 2007). Furthermore, it has been reported that a higher training frequency is advantageous for muscle strength if an increased frequency also increases the training volume (Grgic et al., 2018). Of note, the same study also reported that a higher training frequency appeared to be favorable for muscle strength especially in women, compared to a lower training frequency (Grgic et al., 2018).

Training frequency can be increased by adding more training days per week or by increasing the number of daily training sessions. The latter, to divide the daily training program into multiple shorter sessions, is frequently used by athletes (Storey et al., 2012) and has shown promising results (Häkkinen and Kallinen, 1994; Hartman et al., 2007). For example, Hartman et al. (2007) examined nationally competitive male weightlifters and reported that performing two shorter sessions per day over a 5-week training period led to superior increases in muscle strength compared to performing one session per day. Therefore, it is possible that shorter sessions may lead to less fatigue and more work performed.

To the best of our knowledge, only one study has compared the acute effects of performing one long vs. two shorter resistance training sessions in 1 day (Bartolomei et al., 2011). Bartolomei et al. (2011) examined the effects of performing eight sets of 10 repetitions (75 s between sets) at 70% of 1-RM in bench press in resistance-trained men. The participants were divided into two groups: one group completed all sets in the same session while the other group split the sets into two shorter sessions (four sets per session). The results showed that the participants were able to complete the same number of repetitions at a higher training intensity (percentage of 1-RM) and had faster recovery rates when performing two shorter compared to one longer session.

How an activity is perceived might have implications as to whether a person chooses to continue with that activity (Ekkekakis et al., 2005; Williams et al., 2008). For example, shorter sessions lead to the use of higher training loads and promote faster recovery (Bartolomei et al., 2011). Furthermore, longer training sessions increase the perception of effort compared to shorter sessions (Fusco et al., 2020). This increased perception of effort may also lead to an increased perception of discomfort, since effort and discomfort have been shown to be related (Steele et al., 2016). If performing a daily training program in multiple short sessions leads to increased performance/training loads, faster recovery and less perception of effort and discomfort, it is also likely that it will be perceived as more pleasurable and enjoyable. Furthermore, the perception of being ready to train should be greater the following day.

Another argument for dividing the workout into shorter sessions is the aspect of time. Time is one of the most reported barriers for engaging in exercise (Hoare et al., 2017; Hurley et al., 2018) and having time to complete long workouts may be difficult. However, conducting two short sessions throughout the day may be more manageable and therefore be perceived as more pleasurable, enjoyable and preferable than one longer session. Importantly, no previous study has examined the perception of performing one long or two shorter resistance training sessions in 1 day.

Considering this gap in the literature, the aim of this study was to compare the acute effects of performing a lower body resistance-training program in one long or two shorter sessions in 1 day on training volume and different affective measures. We hypothesized that two shorter lower-body resistance-training sessions would lead to greater perceived pleasure, enjoyment, readiness to train and training volume, and lower perceived effort and discomfort, compared to one long session among resistance-trained women. Consequently, we also expected most participants to prefer dividing the workout into two shorter training sessions.

## Materials and methods

### Study design

**Figure 1:**
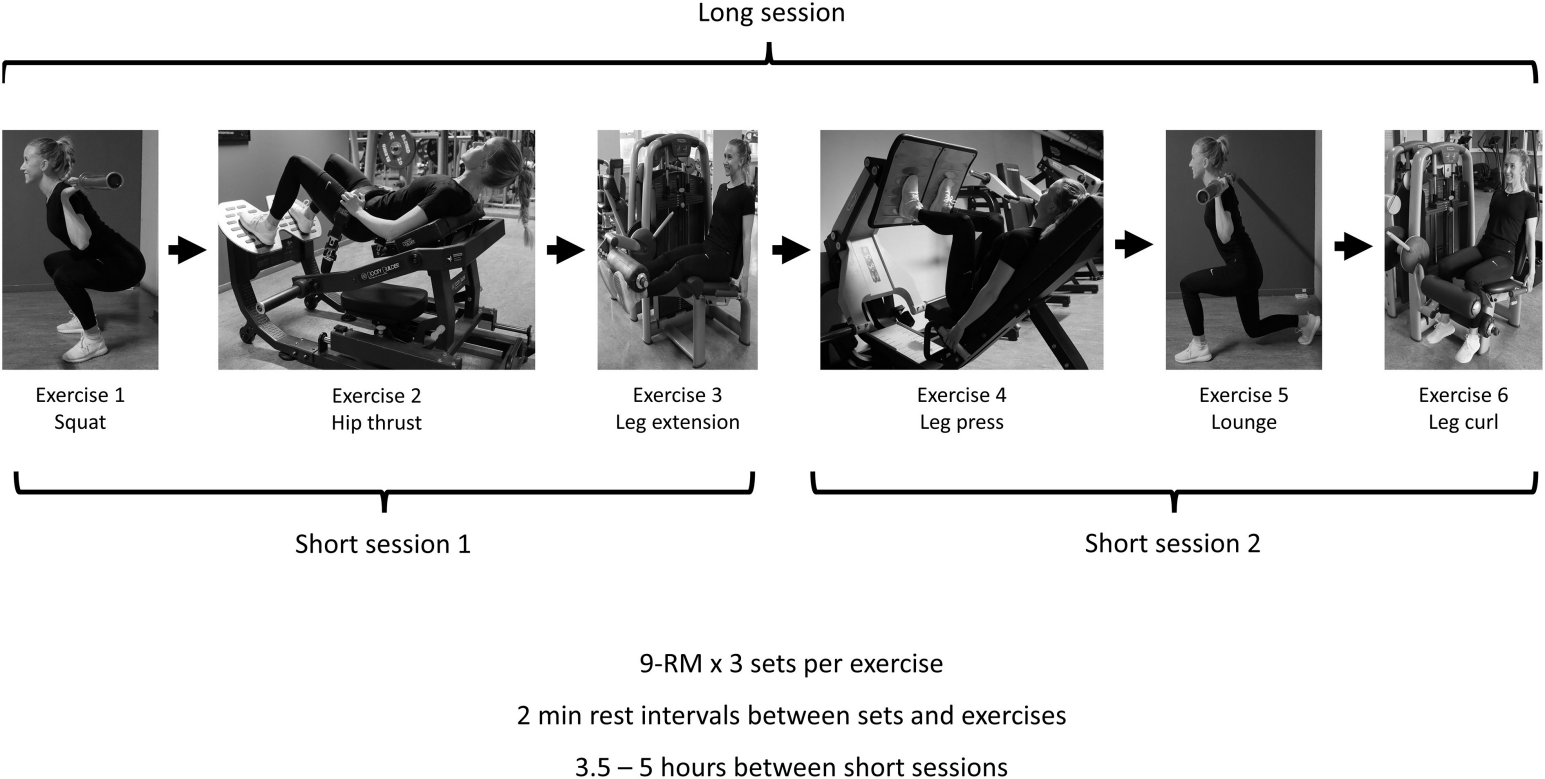
An overview of the long and the two short sessions.

We employed a within subject crossover design to compare the training volume and the affective responses from one long vs. two shorter resistance-training sessions for the lower body. The order of the sessions was randomized and counterbalanced. A familiarization session was conducted in advance of the two experimental sessions. The long session consisted of six exercises (in the order they were conducted: the squat, hip thrust, leg extension, leg press, lunge, and leg curl) focusing on the hip and thigh muscles. The same exercises, and exercise order, were performed in the two short sessions, three exercises per session (see Figure 1 for an overview of the different sessions). The two sessions were divided by a rest period of 3.5–5 h. Training volume, defined as repetitions per session (Haff, 2010; Scott et al., 2016) (using ~9 repetition maximum (RM) weights), and training duration was recorded during all sessions. Number of repetitions was used since all other intra-exercise variables (Coratella, 2022) were held constant between the different sessions. Ten minutes after completion of the sessions, participants were asked how they perceived the session related to effort, discomfort, pleasure/displeasure and enjoyment. Twenty-four hours after each session the participants were contacted through phone and asked about their readiness to train. Forty-eight hours after the last session, participants were contacted by mail asked which of the two sessions they would use as their regular training routine, and the main reason for their choice.

### Participants

Twenty-three women with 3.9 ± 1.9 years of resistance-training experience were recruited to participate in the study. Their mean ± SD characteristics were; age: 22± 2 years, body mass: 66.4 ± 7.5 kg, stature: 166 ± 6 cm, self-reported 1-RM squat: 84 ± 19 kg, and self-reported 1-RM deadlift: 92 ± 20 kg. The sample size was justified performing a priori power analysis in SPSS (IBM Corp. Released 2020. IBM SPSS Statistics for Windows, Version 27.0. Armonk, NY: IBM Corp) based on the difference in training load between the groups observed in Bartolomei et al. (2011) (mean ± standard deviation; 59.9 ± 6.5 vs. 48.7 ± 5.3%), an alpha level of 0.5, Pearson product-moment correlation of 0.5 and power of 0.8. Participants were recruited through posters, personal invitations, meetings, and social media. To be included in the study the participants had to be 18–30 years old, have more than 2 years' experience with resistance-training, be familiar with and able to perform the exercises with good technique, and not have any injuries that prohibited maximal exertion. The participants agreed to refrain from alcohol and training of the lower body 48 h in advance of each session. Furthermore, they were asked to avoid all forms of physical training between the two short sessions or within 24 h after the sessions. They were informed orally and in writing about the procedures and provided a written consent before being enrolled in the study. The procedures were processed by the Norwegian Center of Research Data (ref nr 170233) and were conducted according to the Declaration of Helsinki and the ethical guidelines set by the University College‘s institutional review board.

### Procedures

In the familiarization session we assessed participants' anthropometrics and defined the individual standardization and the training load for each exercise (~9-RM). The 9-RM loading was chosen because it is in the middle of the 6–12RM range recommended for resistance-trained individuals (Garber et al., 2011). Since the participants were experienced with the specific exercises and equipment, they self-reported their 9-RMs. If they were unsure of their 9-RM, they performed progressive sets in that specific exercise until they were able report a specific load. Importantly, regardless of whether 9-RMs were entirely accurate, the same loading was used in all sessions. We also introduced participants to the different affective scales in the following order: subjective experience of the sessions in terms of effort, discomfort, pleasure/displeasure and enjoyment (see measurements for more details).

The first training protocol was performed 3–5 days after the familiarization session and the two training protocols (long session vs. two brief sessions) were separated with 4–6 days. Before each session, the participants conducted a standardized warm-up consisting of 5 min cycling on a low intensity (Borg's RPE scale: 10–11) and two sets of squats lifting nine repetitions at 50% of the self-reported 9-RM. The rest interval between each set was 2 min.

The experimental sessions consisted of three sets per exercise using the same relative load (9-RM) and a rest interval of 2 min between sets and exercises. The only difference between the sessions was that in the long session all exercises were performed consecutively while in the two short sessions the first three and the last three exercises were divided by a rest period of 3.5–5 h.

The participants were instructed to have an external focus (i.e., focusing on moving the weight and not on using the muscles) and perform repetitions to failure in each set, where failure was defined as not being able to lift the weights throughout the range of motion or perform another repetition with proper technique. The repetitions were performed continuously (eccentric-concentric movement) in the participants self-selected/normal, but controlled tempo (i.e., always controlling the weights, no cheating allowed). The same two test leaders were present in all sessions to ensure that the execution of the repetitions (within and between the sets and sessions) was as identical as possible and that the standardizations from the familiarization session was used. An overview of the standardization and range of motion of the different exercises is presented in Table 1. Furthermore, the test leaders counted the repetitions in each set, kept track of time and presented the affective scales to the participants. To keep the sessions as similar to a regular training session as possible, the test leaders did not interfere with the lifting (e.g., spotting, motivating, giving feedback etc.). No inter-set rest was allowed. Since all other intra-exercise variables were held constant (Coratella, 2022), the total number of successful repetitions performed were used as a measurement of the training volume (Haff, 2010; Scott et al., 2016).

**Table 1:** Description of standardizations and execution of the different exercises.

**Exercise**	**Standardization**	**Execution of the reps/ range of motion**
Squat	Barbell resting on the upper trapezius. Shoulder width between the feet Back kept in normal position throughout the lift	Eccentric: Descending from the extended position until the femur is parallel to the floor Concentric: Ascending until the hip- and knee joints are extended.
Hip thrust	Belt resting on the hip Arms resting on the hip Shoulder width between the feet	Eccentric: Descending from the extended position until the plates touch the floor Concentric: Ascending until the hip joint is extended.
Leg extension	Bottom and back in contact with the chair. Arms gripping the handles Footpad resting just above the ankle joint	Eccentric: Descending from the extended position until the plates touch the stack Concentric: Ascending until the knee joints are extended.
Leg press	Bottom and back in contact with the chair at all times. Arms gripping the handles Shoulder width between the feet	Eccentric: Descending from the extended position until a 90-degree angle in the knee joints Concentric: Ascending until the knee joints are extended.
Lunge	Barbell resting on the upper trapezius Shoulder width between the feet Back kept in normal position throughout the lift Same step length in all repetitions	Eccentric: Stepping forward and descending from the extended position until the knee of the back leg is touching the floor Concentric: Ascending and stepping backward until the hip- and knee joints are extended and feet parallel.
Leg curl	Bottom and back in contact with the chair. Arms gripping the handles Footpad resting toward the Achilles tendon	Eccentric: Ascending from the flexed position until the knee joints are extended. Concentric: Descending until 90-degree angle in the knee joints.

### Measurements

#### Affective measures

The perception of the different sessions was assessed through four different scales. None of the participants had any previous experience with the scales. The scales were presented to the participants 10 min after completing the last set. The participants were asked to consider the whole session when giving their answers. All scales were shown to the participants while the test leader read the question which was also included above the scales. The mean of the answers from the two short sessions was used in the analysis. Prior to the study, the scales were translated from the original forms to Norwegian. The scales were translated independently by three of the authors (AHS, HP and VA). The three translations were then compared, discussed, and agreed upon the final versions. A professional translator translated these versions back to English which were compared with the originals. In general, there were only minor differences between the versions, which were adjusted after mutual agreement.

The perception of exertion was differentiated into effort and discomfort (Steele et al., 2016). Effort was measured using the rating of perceived exertion for effort scale (Borg CR-10 RPE), while discomfort was measured using the rating of perceived exertion for discomfort scale (RPD) (Fisher and Steele, 2017). Both scales consist of 11-items and ranges from no effort/discomfort to maximal effort/discomfort. Based on a previous recommendation (Halperin and Emanuel, 2020), the RPE scale was presented to the participants with the following phrase: “*How much of your perceived physical capacity out of your perceived maximum (10 being your maximum) did you invest to complete this workout?*”. The RPD scale was presented with the following phrase: “*Based on the completed session, how much discomfort did you feel? The scale ends at 10 which could be described as you could not imagine the sensations relating to physical activity being any more intense?”* (Steele et al., 2016). The upper and lower limit were anchored by the following sentence “*0 can be described as feeling no noticeable sensation relating to the training while 10 would be the most intense training related sensation you could imagine*.” Both scales have shown acceptable reliability (Steele et al., 2016).

The perceived pleasure/displeasure with the session was measured using the session pleasure/displeasure feelings scale (sPDF). The scale is a bipolar 11-point scale stretching from −5 (very bad) to 5 (very good), where 0 is considered neutral and have previously shown good reliability (Unick et al., 2015). The sPDF scale was presented with the following phrase: “*How was your workout?*” (Ribeiro et al., 2019). The upper and lower limits were anchored by the following sentence: “*-5 can be described as perceiving the session as one of the worst/least pleasurable training sessions you have ever conducted while 5 would be one of the best/most pleasurable training sessions you have ever conducted.”*

How much the participants enjoyed the sessions was measured using the exercise enjoyment scale (EES). The scale ranges from one to seven with one being “not at all” and seven being “extraordinary.” The scale was presented with the following question: “*How much did you enjoy the exercise session?*” (Schwartz et al., 2021). The upper and lower limit were anchored by the following sentence: “*1 can be described as perceiving the session as one of the least enjoyable training sessions you have ever conducted while 7 would be one of the most enjoyable training sessions you have ever conducted.”* The EES scale has been reported to be valid (Stanley et al., 2009).

Forty-eight hours after the last session, the participants were contacted by e-mail and asked the following questions: “*If you had to choose one of the two training protocols (one long or two short sessions) as your regular training schedule, which would you prefer, and what is the main reason for this choice?*”. The participants answered by replying to the mail. The answers were aggregated and grouped based on the underlying theme of the explanation.

#### Readiness to train

To evaluate how the different sessions affected the perception of training ability, the participants were contacted 24 h after long session and 24 h after the median of the two short sessions to answer a questionnaire regarding readiness to train (Lombard et al., 2021). The questionnaire consisted of seven questions with responses made from bipolar scales ranging from 1 to 4, 1 to 5 and 1 to 10. The upper and lower limit were anchored by the following sentence: “*1 can be described as not at all/extremely low and 4, 5, 10 (depending on lower/upper end of the scale) can be described as extreme amount/extremely high.”* The questions were: Q1“*Do you feel physically strong today?”*, Q2 “*Do you feel mentally strong today?”*, Q3 “*How would you describe your health today?”*, Q4 “*How would you describe your appetite today?”*, Q5 “*How would you describe your sleep quality over the past 24 h?”*, Q6 “*Do you have any muscle soreness today?”* and Q7 “*Rate your motivation to train today”*. The questionnaire has not been validated; however, it has shown to be more sensitive to fatigue than objective measures such as performance in countermovement jump (Lombard et al., 2021).

### Statistical analysis

The Shapiro-Wilk test was used to assess normality in the continuous variables training duration and training volume (number of reps). Training duration was found to be normally distributed (*p* = 0.06–0.15) while training volume was not (*p* < 0.01). Paired *t*-tests were therefore used to assess differences in training duration while the Wilcoxon signed rank test was used to assess differences for training volume and the ordinal variables (RPE, RPD, sPDF, EES and readiness to train questionnaire). The results for the ordinal variables are presented as median (interquartile range) while the other variables are presented as means ± standard deviations. Cohen‘s d effect size (ES) was calculated for the continuous variables using the following equation: mean pre–mean post divided by the pooled standard deviations of the two. An effect size of 0.2–0.49, was considered small, 0.5–0.79 medium and ≥ 0.8 large (Cohen, 1998). For the ordinal data effects size was calculated as product-movement r using the following equation: r = z/√n, with z being the z-value of the Wilcoxon signed ranked test and n being the number of participants. A product-movement r of 0.1–0.29 was considered small, 0.3–0.49 medium and ≥ 0.5 large (Cohen, 1998). Statistical significance was accepted at *p* < 0.05. The statistical analyses were performed using SPSS (IBM Corp. Released 2020. IBM SPSS Statistics for Windows, Version 27.0. Armonk, NY).

## Result

The median RPE and RPD was on average 1 point higher (RPE; Z = −3.536, *p* < 0.001 and RPD; Z = −2.022, *p* = 0.043, Table 2) in the long compared to the short sessions. Furthermore, the sPDF was also rated higher in the long session compared to the short session (Z = −2.589, *p* = 0.010) while there was no difference between the sessions for EES (Z = −1.565, *p* = 0.118).

**Table 2:** Affective measures and readiness to train for the long and the short sessions [median (interquartile range)].

	**Long session**	**Short sessions**	***P*-value**	**Effect size**
**Affective measures**				
RPE (0–10)	7 (1)	6 (2)^*^	<0.01	0.73
RPD (0–10)	6 (2)	5 (2)^*^	0.04	0.42
sPDF (−5–5)	4 (2)	4 (1)^*^	0.01	0.54
EES (1–7)	5 (1)	5 (1)	0.12	0.33
**Readiness to train**				
Do you feel physically strong today? (1–5)	3 (0)	3 (1)	0.57	0.11
Do you feel mentally strong today? (1–5)	3 (0)	3 (1)	0.86	0.04
How would you describe your health today? (1–4)	3 (0)	3 (1)	0.20	0.27
How would you describe your appetite today? (1–5)	3 (2)	3 (1)	0.17	0.29
How would you describe your sleep quality over the past 24 h? (1–4)	3 (1)	3 (1)	0.84	0.04
Do you have any muscle soreness today? (1–10)	3 (2)	2 (2)	0.34	0.20
Rate your motivation to train today (1–10)	3 (1)	3 (2)	0.70	0.08

* = different from long (p < 0.05), RPE, rate of perceived exertion effort; RPD, rate of perceived exertion discomfort; sPDF, session pleasure/displeasure; EES, exercise enjoyment.

The training volume was 3% higher in the two short sessions compared to the long session (169 ± 11 vs. 164 ± 8 repetitions, p = 0.002, ES = 0.42, Table 3) while the training duration was 7% shorter in the short sessions (43 ± 2 vs. 46 ± 4 min, *p* = 0.005, ES = 0.89).

**Table 3:** Accumulated number of repetitions for three sets using ~9RM loading for each exercise in the long and short sessions (mean ± standard deviation).

	**Squat**	**Hip thrust**	**Leg extension**	**Leg press**	**Lunge**	**Leg curl**	**Total**
Long	27 ± 2	27 ± 2	28 ± 3	27 ± 1	27 ± 1	27 ± 2	164 ± 8
Short	27 ± 2	27 ± 1	29 ± 3	28 ± 2	28 ± 3	30 ± 4	169 ± 11^*^

* = different from long (p < 0.05).

Twenty-four hours after the sessions there were no differences between the protocols in the perception of readiness to train (Z = −1.565– −0.182, *p* = 0.166–0.856, Table 1). When asked 48 hours after the last session which session they preferred as their regular training routine, 22 chose the long session while one participant chose the two short sessions.

## Discussion

This is the first study to examine the acute perceptive effects of performing one long or two shorter resistance training sessions in 1 day. In agreement with our hypothesis, the results showed that in resistance-trained women performing one long session led to a greater perception of effort and discomfort and a reduced training volume compared to two shorter sessions. However, in contrast to our hypothesis, the long session was perceived as more pleasurable than the two short sessions. Furthermore, there was no difference in session enjoyment and readiness to train between the two protocols. When asked about which session they preferred, all except one participant preferred the long session.

A possible explanation for the increased training volume when performing two short sessions is that performing briefer and more frequent sessions allows for more intense sessions and thereby lifting more repetitions per set due to less accumulated fatigue (Hartman et al., 2007). Importantly and surprisingly, the difference was only three percent, and the effect size of this finding was considered small (ES = 0.42). Therefore, the importance of this finding is debatable, especially for the general population, although it might be of relevance for competitive strength training athletes as the cumulated effect can lead to a modest, but practically meaningful increase in training stimuli over time. Of note, the population in the present study was resistance-trained and may therefore be able to sustain the number of repetitions over a relatively long session. Although speculative, the difference might have been greater with a less experienced population.

Although the difference in repetitions became more evident as the sessions progressed, suggesting that the accumulated work ultimately resulted in fatigue and thus a decrement in performance, it was expected that the reduction in repetitions per set should be higher, especially in the long session. Importantly, in some of the exercises (squat and deadlift) there may be more of a technical failure (not able to keep a straight back etc.) compared to an absolute failure (the neuromuscular system not able to lift the weights one more time), which may have affected the ability to keep the number of repetitions relatively stable throughout the session. Further, the participants were not spotted during the lifts which, due to safety reasons, may have affected number of repetitions and consequently fatigue before aborting the set. However, these factors were kept identical in all sessions. As mentioned, the difference in volume emerged at exercises 3–6 in the long session (Table 3). Consequently, it would be interesting to compare a more extensive training program to observe if the difference becomes more apparent. Our finding is in accordance with Bartolomei et al. (2011), who compared performing eight sets of bench press in one or two sessions. In opposition to our design, they kept the number of repetitions constant and compared the training intensity (percentage of 1-RM). The authors reported a mean loading intensity of 59.9% of 1-RM when dividing the sets into two sessions compared to 48.7% when performing all sets in the same session.

As hypothesized, both the perception of effort and discomfort were increased after the long compared to the two short sessions. When increasing the work and/or work rate, the metabolic and endocrine stress responses also increase (Paz et al., 2017; Weakley et al., 2017). Further, accumulation of different metabolic factors has been shown to increase the sensation of fatigue and pain/discomfort (Pollak et al., 2014) and may therefore explain our results. Notably, effort and discomfort are different perceptions, however, they are reported to be related (Steele et al., 2016), and the increased perception of effort and discomfort observed in our study could, at least partly, explain each other.

Contrary to our hypothesis, the long session was considered more pleasurable despite being perceived as more strenuous and discomforting. The difference in session pleasure may be related to the resistance-trained sample who therefore may enjoy sessions that are perceived as more discomforting and more demanding in regard to effort. This speculation is supported by the fact that 22 out of 23 reported to prefer the long session. Furthermore, the feeling of an intense workout, together with time-efficiency was the most common explanations for that choice. Importantly, time-efficiency is most likely related to the surroundings of the training (not having to travel to the training center twice in a day), and not the training *per se*.

Our finding is in line with the results from a previous study from our lab comparing traditional and superset resistance training among resistance-trained (Andersen et al., 2022). The study indicated that the more strenuous superset session led to higher discomfort and effort among participants, but also tended to be more pleasurable (Andersen et al., 2022).

There was no difference between the two protocols in the perceived readiness to train 24 h after the sessions. Although, the training volume difference was statistically significant, the difference was rather small (3%). Therefore, the difference might have been too small to induce differences in the perception of readiness to train 24 h after the sessions. Our finding differs with that of Bartolomei et al. (2011) reported a faster recovery process when dividing the total training volume into two shorter sessions. Importantly, Bartolomei et al. (2011) measured the recovery process by objective measures, such as isometric bench press and power output in the bench press, which could explain the different findings between the studies. Of note, Bartolomei et al. (2011) did not report any difference in muscle soreness between the protocols 24 h after the sessions.

The study has some limitations that should be considered when drawing practical conclusions. Only resistance-trained women were recruited to this study and the findings therefore may not necessarily be generalizable to other populations. Further, only exercises for the lower body were included in the sessions. The findings may have been different if a greater training volume had been implemented, either by including the upper body or increasing the number of exercises/sets for the lower body. Importantly, we wanted the sessions to have a high ecological validity. Therefore, we designed the program as a typical split workout routine and used a total training volume of 18 working sets for the thigh- and hip muscles, which is relatively high. Although the different scales were presented to the participants in the familiarization session, they were not familiar with them prior to the study and the scores might have been different if they had had more experience with the scales. Importantly, the order of the sessions was randomized and counterbalanced so a potential familiarization effect from the first to the second training protocol would have been evened out. Also, the measures were only assessed after the sessions. It has been shown that people are more positive toward training after the exercise (affective rebound effect) (Ekkekakis et al., 2011). Therefore, the perception of the sessions may have changed throughout the sessions. The load used in the present study was the participants self-reported 9-RM. Consequently, the load lifted may not be their true 9-RM. However, as shown in Table 3, the reported load was close to the number of repetitions actually lifted. Importantly, the same load was used in all sessions to allow for comparisons between the different training programs. Finally, menstrual cycle and nutritional intake was not controlled in the study, which may have influenced the results.

From a practical point of view, splitting the lower body workout into two shorter intra-day sessions produced favorable increases in training volume and reductions in perceived rating of effort and discomfort in resistance-trained women. However, the long session was perceived as more pleasurable and 96% (22 out of 23) of the participants preferred the long session for their normal training routine. The two main reasons for this choice were time-efficiency, i.e., don‘t have to go to the gym twice, and an appreciation of the feeling of having performed a hard/exhausting session. Regarding time-efficiency, for some people it can be easier to schedule two short training sessions rather than one longer session depending on proximity to training facilities and individual time schedules. Thus, from a time-efficiency point of view, and in support of previous findings (Iversen et al., 2021), people should choose between performing one long or two shorter sessions in 1 day depending on what suits their individual calendar and preferences. Importantly, the different conditions did not affect the participants‘ readiness to train more than the other. Although these findings may be exclusive for the sample of the present study (resistance-trained women), it implies that there are several factors that should be considered when designing resistance training programs.

In conclusion, performing a longer, lower body resistance-training session led to greater perceptions of effort, discomfort and session pleasure than splitting the same program into two shorter sessions in resistance-trained young women. However, two shorter intra-day sessions led to modestly higher training volume, defined as number of successful repetitions.

## Data availability statement

The raw data supporting the conclusions of this article will be made available by the authors, without undue reservation.

## Ethics statement

Ethical review and approval was not required for the study on human participants in accordance with the local legislation and institutional requirements. The patients/participants provided their written informed consent to participate in this study. Written informed consent was obtained from the individual(s) for the publication of any potentially identifiable images or data included in this article.

## Author contributions

HP and VA came up with the idea and wrote the first draft. All authors helped in developing the methodology, contributed to the article, and approved the submitted version.

## Conflict of interest

Author BS serves on the scientific advisory board of Tonal Corporation, a manufacturer of fitness equipment. The remaining authors declare that the research was conducted in the absence of any commercial or financial relationships that could be construed as a potential conflict of interest.

## Publisher's note

All claims expressed in this article are solely those of the authors and do not necessarily represent those of their affiliated organizations, or those of the publisher, the editors and the reviewers. Any product that may be evaluated in this article, or claim that may be made by its manufacturer, is not guaranteed or endorsed by the publisher.
